# Book Review: Herbal Principles in Cosmetics: Properties and Mechanisms of Action

**DOI:** 10.3389/fphar.2019.01513

**Published:** 2020-01-09

**Authors:** Marco Nuno De Canha, Aimee Steyn, Analike Blom van Staden, Bianca Daphne Fibrich, Isa Anina Lambrechts, Lilitha Lwando Denga, Namrita Lall

**Affiliations:** ^1^Department of Plant and Soil Sciences, University of Pretoria, Pretoria, South Africa; ^2^School of Natural Resources, University of Missouri, Columbia, MO, United States; ^3^College of Pharmacy, JSS Academy of Higher Education and Research, Mysuru, India

**Keywords:** cosmeceuticals, monographs, mechanism of action, skin care, natural actives

This book forms part of the series Traditional Herbal Medicines for Modern Times, and includes cosmeceuticals used in traditional Chinese medicine, Japanese Kampo medicine, and Indian Ayurvedic practices (Burlando et al., 2010). Consumer demand for cosmetic ingredients from natural sources, with comparable efficacy and safety features of synthetic ingredients, is becoming progressively more popular. In the past, cosmetics were restricted to aesthetic and decorative applications; however, modern cosmeceuticals are defined as cosmetics with the ability to alter skin functionality and improve health, eliminating any ambiguity between cosmetology and dermatology (Taofiq et al., 2018).

Chapter 1 of this book describes skin morphology, cell types, and structural features responsible for maintaining healthy skin. Chapter 2 focuses on the major phytochemical classes, their synthesis in plant cells, and their medicinal uses. Briefly, fatty acids and triglycerides act as emollients and emulsifiers providing the skin with moisture and adding to the waterproofing effect by interacting with the lipophilic fraction of the skin. Terpenoids commonly found in the essential oils of plants have preservative effects due to their ability to cause damage to prokaryote cell walls. They are often also used as antioxidants and for flavoring. Phenolic compounds from plants act as UV filters due to the presence of benzene ring structures. Alkaloids such as vincristine and vinblastine act as chemotherapeutic agents by affecting microtubule formation in cancer cells. Carbohydrates such as glucans act as skin moisturizing agents by preventing epidermal water loss. O-glycosides are cardioactive compounds that can prevent heart failure by stimulating the sodium/potassium ATPase pump. Hydroxy acids increase skin desquamation and exfoliate the skin for cell renewal. Chapter 3 provides valuable information to potential formulators searching for emulsifiers, humectants, and emollients from natural sources by arranging the plant species alphabetically using their common names. It includes herbal preservatives and agents enhancing skin penetration. Valuable insights into the side effects of topical formulations, even of those containing natural ingredients and their role as comedogenic and acnegenic agents, are mentioned in this section.

Chapter 4 of this book comprises of 70 plant species summarized in the form of monographs. These provide the reader with a botanical description, region of origin, phytochemical constituents, and their therapeutic properties, as well as their current dermatological and cosmetic uses. They also include a section outlining the side effects and toxicological data associated with the use of these extracts.

Several well-known plant species including *Aloe vera*, *Argania spinosa* (argan), *Azadirachta indica* (neem), *Ginkgo biloba* (gingko), and *Moringa oleifera* (Moringa) are described. These species are used extensively as active ingredients in cosmetic products.

Numerous species used as flavor enhancers in cuisine are also described. *Cinnamomum verum* (cinnamon) containing cinnamaldehyde is largely used in cosmetics for its fragrance, most commonly in the form of an oil. *Rosmarinus officinalis* (rosemary), high in caffeol derivatives such as rosmarinic acid, is used to treat acne by reducing swelling. Rosemary essential oil is common in perfumery and hair care. Hops (*Humulus lupulus*), popular in beer flavoring and preservation, contains several constituents including α- and β-acids and prenylnaringenin, contributing to the photoprotecting and regenerative activity of this species. The multi-purpose hydrodistilled oil of lemon balm (*Melissa officinalis*) used as a perfuming, antimicrobial, and antiviral agent is said to be due to the presence of citronellal.

Edible berries including those from *Sambucus nigra* (European elder), *Vaccinium myrtillus* (bilberry), and *Vitex agnus-castus* (chasteberry) are used for various disorders associated with the skin and are often used to protect the skin from the harsh effects of the sun.

The versatility of various plant parts of common fruit producing species including *Mangifera indica* (mango tree) and *Punica granatum* (pomegranate) are discussed. They are rich in bioactive phenolic compounds. Mangiferin and ellagic acid are highlighted in the monographs of these two species. The fruit and seeds of *Cocos nucifera* (coconut palm) is used in several cosmetic formulations and soaps for its hydrating and regenerating properties attributed to the compound, lauric acid. Olive oil, produced from the fruit of *Olea europaea*, has hydrating, photoprotective, and soothing effects on the skin. Interestingly, species like *Plukenetia volubilis* (Inca peanut) and *Lentinula edodes* (shiitake mushroom) contribute to enhancing skin barrier function and hydration due to the presence of essential lipids and amino acids. Similarly, *Sclerocarya birrea* (marula) fruits and seeds are rich in vitamin, minerals, and proteins. Red grape (*Vitis vinifera*) extracts rich in polyphenols, including resveratrol, are widely used to improve skin health and protect from UV radiation. *Macadamia integrifolia* (macadamia nut) oil high in palmitoleic acid is used as a natural preservative of cosmetic ingredients, inhibiting Gram-positive bacterial growth. *Glycyrrhiza glabra* (licorice) mimics cortisol activity and is, therefore, used for inflammatory skin disorders.

Alkaloid compounds produced in well-known species are discussed, with a particular focus on caffeine, theobromine, and theophylline. *Coffea arabica* (coffee plant), *Cola acuminata*, and *Cola nitida* (Cola) are producers of caffeine while *Camellia sinensis* (green tea) and *Theobroma cacao* (cocoa plant) produce theobromine and theophyllines. *Paullinia cupana* (guarana) produces all three of these compounds. In general, these compounds show lipolytic activity, with the cosmetic potential to treat cellulite.

*Plectranthus barbatus* (Indian coleus), a forskolin producing species, is also used in cellulite treatment; however, it has several other applications including acne, psoriasis, and oral hygiene products. *Trichilia emetica* (mafura) seed oil forms a butter at room temperature. This butter is used to moisturize skin and improve wound healing and skin health due to the presence of essential fatty acids. The oil of *Aesculus hippocastanum* (horse chestnut), containing the complex saponin escin, is used to sooth erythrema caused by inflammatory skin disorders.

The book highlights several algal species used in cosmetics. *Fucus vesiculosus* and *Laminaria digitate* and *Undaria pinnatifida* are all brown alga. These organisms are high in polysaccharides alginic acid, for example, which can be used as a thickening and emulsifying agent in cosmetic formulations. These polysaccharides are also emollients and increase skin hydration, preventing ageing. These polysaccharides are also in *Palmaria palmata*, a red algae. *Lithothamnion corallioides* and *Phymatolithon calcareum* (Maërl) are calcareous red algae used as exfoliants for skin rejuvenation. *Chlorella vulgaris* (green algae) has anti-ageing effects through metalloproteinase inhibition and cell regenerating properties by stimulating collagen synthesis. Two mosses, *Cetraria islandica* (Iceland moss) and *Chondrus crispus* (Irish moss), are also discussed, which have similar cosmetic applications to the algal species and act as skin hydrating agents and emollients.

Perhaps one of the major criticisms of this book is the lack of clarity of whether the cosmetic and dermatologic use of these herbal principles is based on their use in commercially available products or whether it is solely based on *in vitro* and *in vivo* data or records of traditional uses. From this book review, the authors would like to suggest that subsequent published books of this nature include cosmetic products where these ingredients are being used as the active ingredient and perhaps some form of clinical data to support the use of these herbal principles.

The monograph section of this book covered most cosmetic uses and included applications in skin care, hair care, oral care, as well as functional cosmetic ingredients with potential pharmaceutical applications. The authors successfully keep readers interested by describing actives from plant, algal, and fungal species.

## Author Contributions

All the listed authors have made a direct, substantial, and intellectual contribution to the work and have approved it for publication. MDC edited and submitted the manuscript.

## Conflict of Interest

The authors declare that the research was conducted in the absence of any commercial or financial relationships that could be construed as a potential conflict of interest.
